# Design and Fabrication of a Chitosan-Based Diaphragm Digital Stethoscope for Heart Sound Acquisition

**DOI:** 10.3390/mi17050555

**Published:** 2026-04-30

**Authors:** María Claudia Rivas Ebner, Seong-Wan Kim, Giyeon Yu, Emmanuel Ackah, Hyun-Woo Jeong, Kyung Min Byun, Young-Seek Seok, Seung Ho Choi

**Affiliations:** 1 Department of Biomedical Engineering, Yonsei University, Wonju 26493, Republic of Korea; 2 Department of Agricultural Biology, National Institute of Agricultural Sciences, Rural Development Administration, Wanju 55365, Republic of Korea; 3 Department of Biomedical Engineering, Eulji University, Seongnam 13135, Republic of Korea; 4 Department of Electronics and Information Convergence Engineering, Kyung Hee University, Yongin 17104, Republic of Korea; 5 Gangwon-do Agricultural Product Registered Seed Station, Chuncheon 24410, Republic of Korea; air5738@korea.kr; 6 Department of Integrative Medicine, Yonsei University College of Medicine, Seoul 06229, Republic of Korea

**Keywords:** digital stethoscope, chitosan diaphragm, cardiac auscultation, phonocardiogram, wireless biosensing

## Abstract

Cardiac auscultation remains a widely used non-invasive method for assessing cardiac function; however, conventional acoustic stethoscopes are limited by subjective interpretation and lack of digital signal-handling capabilities. This study presents the design and fabrication of a chitosan-based diaphragm digital stethoscope using a biopolymer-derived acoustic interface. Chitosan was extracted from mealworm larvae shells through sequential chemical processing and subsequently processed into a glycerol-plasticized film via solution casting to obtain a flexible diaphragm. The mechanical properties of the diaphragm were evaluated to assess its suitability for acoustic applications. The diaphragm was mechanically coupled to a piezoelectric sensor and integrated into a custom 3D-printed chest piece connected to a microcontroller-based acquisition system. Heart sound signals were acquired from four conventional auscultation sites (aortic, pulmonic, tricuspid, and mitral regions). The recorded signals were processed using band-pass filtering, envelope extraction, and time–frequency analysis to visualize waveform morphology and frequency content. The signals obtained exhibited temporal and spectral features consistent with reported phonocardiography characteristics, including identifiable S1 and S2 components. These results demonstrate the feasibility of using chitosan-based diaphragm materials for heart sound acquisition in a digital stethoscope configuration, providing a low-complexity platform for further development of biopolymer-based acoustic sensing devices.

## 1. Introduction

Cardiovascular diseases (CVDs) remain a leading cause of global mortality, accounting for millions of deaths annually [1]. Timely identification of cardiac irregularities is important in clinical practice. Cardiac auscultation is a non-invasive physical examination method for assessing cardiac function, providing information related to cardiac mechanical activity and rhythm through the analysis of heart sounds [2]. The main heart sounds, S1 and S2, are caused by the closing of the atrioventricular and semilunar valves, respectively, and exhibit distinct timing, amplitude, and frequency patterns under different physiological conditions.

Conventional auscultation with acoustic stethoscopes, while clinically used, is constrained by subjective interpretation, operator skill level, and ambient noise, which may affect diagnostic accuracy [3]. In addition, conventional stethoscopes typically lack integrated digital signal processing and storage capabilities, limiting their use in digital signal analysis workflows.

Digital stethoscopes have been developed to address these constraints by integrating electronic sensors, amplification circuits, and signal-processing algorithms to improve heart sound clarity and facilitate objective analysis [4,5]. Advancements in heart sound segmentation, including envelope-based and probabilistic models, have enabled precise identification of S1 and S2 components, thereby assisting in automated heart-rate extraction and the detection of abnormal sounds [5,6]. The integration of wireless microcontrollers and low-power communication protocols has facilitated real-time data streaming and remote cardiac monitoring, thereby augmenting the importance of digital auscultation for telemedicine and point-of-care diagnostics [7,8,9].

Chitosan, a biopolymer derived from the deacetylation of chitin, has been widely studied for biomedical and sensing applications due to its biocompatibility, biodegradability, mechanical flexibility, and tunable physicochemical properties [10,11]. Chitosan-based films and membranes have been investigated in applications such as flexible sensors, wearable electronics, and biomedical interfaces, where their structural adaptability and ease of processing are advantageous [10,11,12,13]. In particular, solution-cast chitosan films can be tailored in terms of thickness, flexibility, and mechanical response through variations in processing conditions and plasticizer content, making them suitable for applications requiring compliant interfaces.

Despite these characteristics, the use of chitosan as an acoustic diaphragm material in digital stethoscopes has received limited attention. The acoustic interface between the chest wall and the sensing device, particularly the diaphragm, plays an important role in the transmission of mechanical vibrations. While prior work in digital auscultation systems has primarily focused on sensor selection and signal-processing strategies, the influence of diaphragm material properties on signal transmission has been less frequently addressed. Conventional stethoscope diaphragms are typically fabricated from synthetic polymers and integrated into rigid chest-piece structures, with material selection often driven by durability, manufacturability, and standardization considerations.

The exploration of alternative diaphragm materials with suitable mechanical properties and potential sustainability advantages remains an open area of investigation. In this context, chitosan-based films offer a material platform that can be evaluated as a compliant acoustic interface within digital auscultation systems. Their combination of mechanical flexibility and film-forming capability makes them suitable candidates for investigating their role in the transmission of low-amplitude physiological vibrations.

To evaluate the feasibility of the proposed system, heart sounds were acquired from the aortic, pulmonic, tricuspid, and mitral auscultation areas, where differences in acoustic waveforms are expected [2,14]. Time-domain waveform visualization, frequency-domain spectral analysis, spectrogram representation, and envelope-based S1/S2 detection were used to examine waveform morphology and frequency content.

This study presents a prototype digital auscultation system based on a chitosan diaphragm and piezoelectric sensing approach. The system integrates a biopolymer-based acoustic interface with a compact signal acquisition configuration for heart sound recording and analysis.

This work describes the design, fabrication, and experimental evaluation of a chitosan-based diaphragm digital stethoscope. Chitosan derived from mealworm biomass was processed into a glycerol-plasticized film to obtain a flexible diaphragm. The diaphragm was mechanically characterized and incorporated into a custom-designed 3D-printed chest piece to enable mechanical coupling with a piezoelectric sensor. The sensor converts chest vibrations into electrical signals for subsequent acquisition and analysis.

## 2. Materials and Methods

### 2.1. Materials

Mealworm shell biowaste was obtained from Gangwon-do Agricultural Product Registered Seed Station, Chuncheon, Republic of Korea for our study. The reagents and chemicals used in this study were of analytical grade to ensure high precision and reliability in experimental outcomes. The acetic acid (CH_3_COOH) of >99.5% purity was obtained from DAEJUNG Chemicals & Metals Co., Ltd, Siheung-si, Republic of Korea. NaOH with >98.0% purity was obtained from OCI Company Ltd., Seoul, Republic of Korea. Glycerol with ≥99.5% purity was bought from DAEJUNG Chemicals & Metals Co., Ltd., Siheung-si, Republic of Korea.

### 2.2. Extraction of Chitosan from Mealworm-Derived Biomass

**Figure 1:**
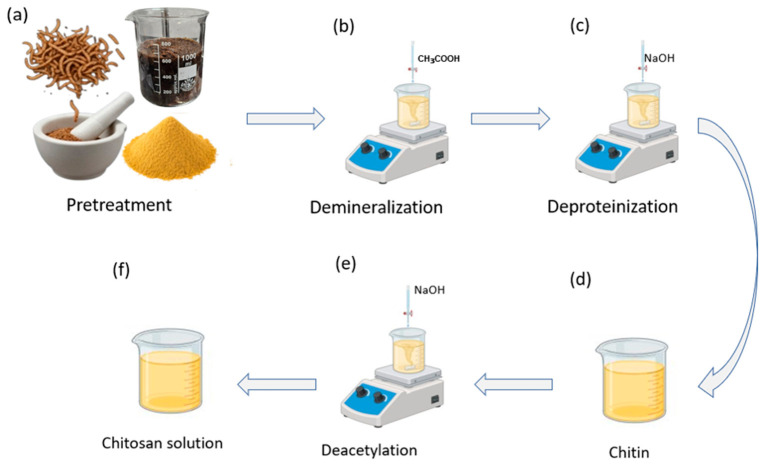
Chitosan extraction from mealworm-derived biomass. (**a**) Pretreatment of dried mealworm residues (drying, grinding, and sieving). (**b**) Demineralization using aqueous acid (CH_3_COOH). (**c**) Deproteination with NaOH. (**d**) Recovery of purified chitin. (**e**) Deacetylation of chitin in alkaline medium (NaOH) to obtain chitosan. (**f**) Preparation of the final chitosan solution for subsequent use.

Chitosan was extracted from mealworm-derived biomass following a sequential acid–alkaline protocol adapted from established insect chitin extraction procedures [15,16]. As illustrated in Figure 1, dried mealworm residues were first ground and sieved to increase surface area and facilitate subsequent chemical treatment (Figure 1a). Demineralization was carried out by immersing the powdered biomass in aqueous acetic acid under continuous stirring to remove inorganic components (Figure 1b).

The demineralized material was subsequently treated with sodium hydroxide solution to remove residual proteins and lipids, yielding purified chitin (Figure 1c,d) [17]. Deacetylation was then performed by exposing the chitin to concentrated NaOH at elevated temperature to convert chitin into chitosan (Figure 1e). The resulting chitosan was thoroughly rinsed to neutral pH and dissolved in dilute acetic acid to obtain a homogeneous casting solution for diaphragm fabrication (Figure 1f).

### 2.3. Fabrication of the Chitosan–Glycerol Diaphragm Film

**Figure 2:**
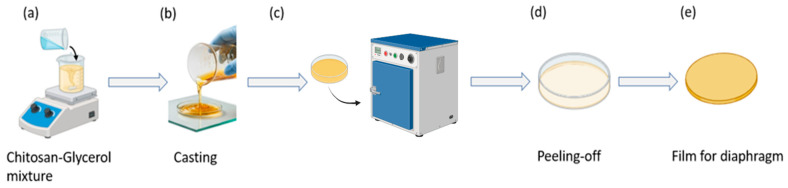
Fabrication process of the chitosan-glycerol diaphragm film. (**a**) Preparation of the chitosan-glycerol casting solution under magnetic stirring. (**b**) Solution casting into a Petri dish. (**c**) Drying in an oven to form a continuous film. (**d**) Peeling-off of the dried film from the Petri dish. (**e**) Freestanding chitosan-glycerol film used as the diaphragm of the digital stethoscope.

Glycerol was incorporated into the chitosan solution as a plasticizer prior to film formation. The mixture was magnetically stirred until a homogeneous solution was obtained (Figure 2a). The resulting casting solution was poured into a Petri dish (Figure 2b) and dried in a convection oven (Lab Companion, Jeio Tech Co., Ltd., Daejeon, Republic of Korea) at 30 °C for 4 h under ambient laboratory humidity conditions (approximately 50% relative humidity) to allow for solvent evaporation and film formation (Figure 2c).

After complete drying, the freestanding film was carefully peeled from the substrate (Figure 2d) and trimmed to approximately 4.3 cm in diameter, consistent with the main diaphragm size of standard clinical stethoscope chest piece, and an average thickness measured using a digital caliper, was approximately 0.2–0.3 mm (Figure 2e). The solution-casting method was selected due to its simplicity and suitability for producing uniform thin films appropriate for diaphragm integration.

### 2.4. Design and Fabrication of the Digital Stethoscope Enclosure

**Figure 3:**
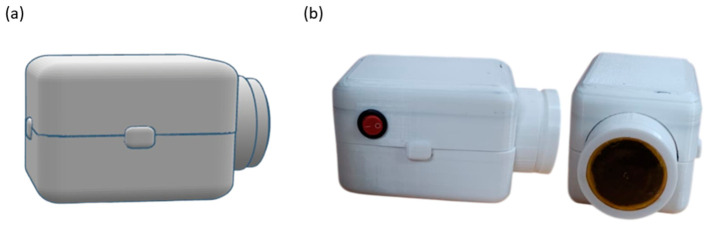
3D-printed enclosure of the digital stethoscope. (**a**) Computer-aided design (CAD) model of the two-part housing with the circular chest-piece port. (**b**) Fabricated 3D-printed prototype showing the assembled device with integrated power switch (**left**) and front view of the chest-piece opening for the chitosan diaphragm (**right**).

The digital stethoscope enclosure was designed using computer-aided design (CAD) software (Tinkercad, Autodesk Inc., San Francisco, CA, USA) to accommodate the diaphragm, piezoelectric sensor, and electronic components within a compact assembly (Figure 3a). The housing consists of a two-part structure that allows for placement of the diaphragm at the chest-facing interface and integration of the sensing and electronic modules (Figure 3b). The enclosure was fabricated using 3D printing to enable rapid prototyping and dimensional customization during the development phase [18].

### 2.5. System Architecture and Signal Acquisition

**Figure 4:**
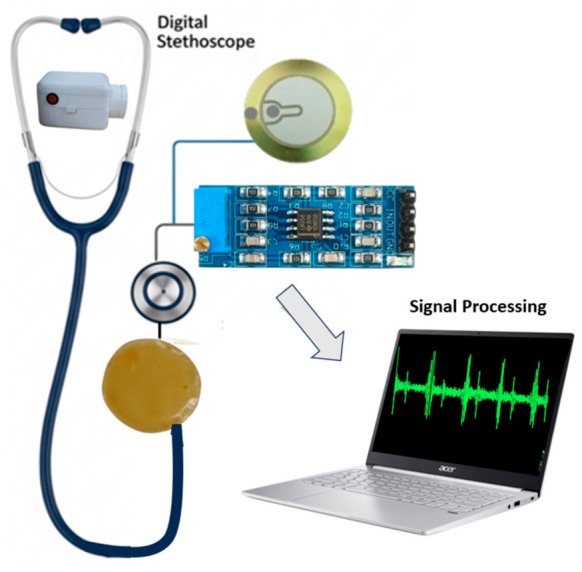
System architecture of the chitosan-enhanced digital stethoscope. A custom diaphragm-integrated piezoelectric sensor captures thoracic acoustic vibrations. The conditioned signal is sent to an ESP32. Heart-sound data are received in MATLAB R2024B for real-time visualization, heart-rate detection, signal analysis, and optional CSV export for further processing.

Figure 4 illustrates the operation of the proposed stethoscope prototype system based on piezoelectric sensing technology. The system consists of a piezoelectric contact sensor (generic, Arduino-compatible, Shenzhen, China) an LM358-based analog preamplifier (Texas Instruments, Dallas, TX, USA), an ESP32 microcontroller unit (Espressif Systems, Shanghai, China) and a host computer (Acer Swift 3, Acer Inc., Taipei, Taiwan) for signal processing and visualization.

A piezoelectric sensor mechanically coupled to the diaphragm converts thoracic vibrations generated by cardiac activity into electrical signals. Piezoelectric transducers are commonly used for detecting mechanical vibrations and acoustic pressure variations and have been widely applied in auscultation-related systems [19].

An LM358 operational amplifier configured as a preamplifier was used to condition the analog output from the piezoelectric sensor prior to digitization. The LM358 was selected due to its single-supply operation and compatibility with embedded systems. The preamplifier stage provides amplitude scaling and impedance interfacing before digital acquisition [20,21].

On the host side, MATLAB was used to acquire and process the PCG signals. Signal processing included digital filtering, time-domain visualization, frequency-domain analysis (FFT and spectrogram), and envelope-based segmentation of heart sound components. Spectrogram representations were computed using short-time Fourier transform (STFT). The spectral power was expressed in a logarithmic decibel (dB) scale and normalized with respect to the maximum amplitude of each recording. This normalization enables consistent visualization and qualitative comparison of acoustic energy distribution across different auscultation sites. Basic peak identification was implemented to assist visualization of cardiac cycles. Processed signals could be exported in CSV format to enable offline analysis and further processing [22,23]. A band-pass filter in the range of 30–150 Hz was applied to enhance the dominant S1 and S2 components while reducing low-frequency motion artifacts and higher-frequency ambient noise. Although certain pathological murmurs may extend beyond this interval, the present work focuses on prototyping and feasibility demonstration rather than comprehensive clinical diagnostic validation.

The overall system integrates a piezoelectric sensing element, analog conditioning stage, and ESP32-based data acquisition, where signal transmission is performed via wired serial communication to a host computer for real-time visualization. The ESP32 microcontroller utilizes an internal 12-bit analog-to-digital converter (ADC) with a 3.3 V reference voltage. Signals were sampled at 200 Hz, which is sufficient to capture the frequency range of interest for heart sounds (approximately 20–200 Hz). Data acquisition was performed via wired USB serial communication to a host computer.

**Figure 5:**
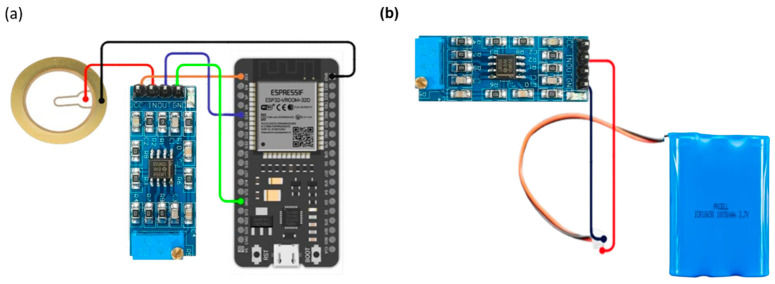
Hardware configuration of the digital stethoscope prototype. (**a**) Connection of the piezoelectric disc sensor to the signal-conditioning (preamplifier) module and the ESP32 development board for the acquisition of heart-sound vibrations; the sensor output is routed through the preamplifier and then into an ESP32 analog input, with a common ground shared by all components. (**b**) Power-supply arrangement showing the 3.7 V single-cell lithium-ion battery wired to the charging/power-management module powering the ESP32-based digital stethoscope.

Figure 5a shows the electrical connections between the piezoelectric sensor, the conditioning circuit, and the ESP32 input interface. A common ground configuration was implemented across components to maintain signal reference stability.

The system was implemented as a laboratory-scale prototype. Power was supplied using a regulated DC source during experimental measurements (Figure 5b) [24].

### 2.6. Auscultation Sites

**Figure 6:**
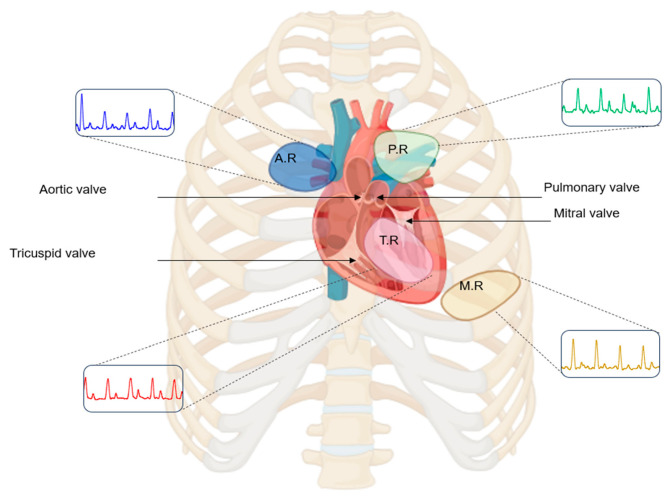
Anterior thoracic view showing anatomical valve positions (shaded) and corresponding auscultation sites: aortic (2nd intercostal space, right sternal border, blue), pulmonic (2nd intercostal space, left sternal border, green), tricuspid (4th intercostal space, left sternal border, red), and mitral (5th intercostal space, left midclavicular line, yellow), with representative waveforms shown at each site.

Heart sounds were acquired from four conventional anterior thoracic auscultation sites: the aortic, pulmonic, tricuspid, and mitral regions (Figure 6). These locations correspond to standard anatomical areas commonly used in clinical auscultation practice, where sounds associated with the respective cardiac valves are typically assessed [25,26]. Recordings were performed while the subject was at rest to minimize motion-related artifacts and physiological variability during acquisition.

## 3. Results and Discussion

### 3.1. Mechanical and Structural Characterization of the Chitosan-Based Diaphragm}

**Figure 7:**
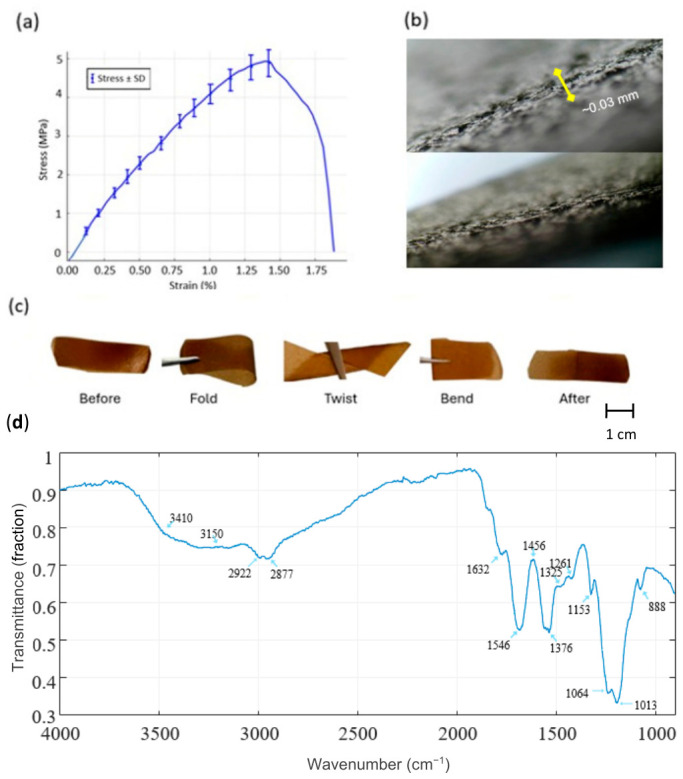
Material characterization of the chitosan diaphragm. (**a**) Tensile stress–strain curve (mean ± SD) demonstrating the mechanical performance of the chitosan diaphragm, including its high elongation and moderate tensile strength. (**b**) Cross-sectional micrograph of the diaphragm, revealing a compact and well-bonded layered structure with no significant cracks or voids, confirming uniform film formation. (**c**) Flexibility assessment of the diaphragm before deformation, during folding, twisting, bending and after recovery, confirming its structural integrity and mechanical resilience. (**d**) FTIR spectrum of the chitosan diaphragm, confirming the chemical structure of chitosan.

Figure 7 illustrates the mechanical, morphological, and flexibility characterization of the chitosan diaphragm. Figure 7a shows the tensile stress–strain curve (mean ± SD). In the initial low-strain region (approximately 0–1% strain), the stress–strain relationship appears approximately linear, consistent with elastic deformation. The Young’s modulus was estimated from the slope of this region using the relation *E* = Δ*σ*/Δ*ε*. Based on the change in stress over the corresponding strain interval, the modulus is estimated to be on the order of ~300–400 MPa, within the range of reported values for solution-cast chitosan films exhibiting mild plasticization and dense hydrogen-bonded networks [27].

Outside the elastic regime, the diaphragm exhibits strain-hardening behavior, with stress increasing as strain increases. The material reaches an ultimate tensile stress of around 4.8–5.1 MPa at a strain of roughly 1.3–1.4%, succeeded by a decrease in stress. Final fracture occurs at roughly 1.7–1.8% strain, signifying a mostly brittle-to-semi-brittle failure mechanism with constrained plastic deformation.

This mechanical behavior is typical of thin biopolymer films, where robust intermolecular hydrogen bonding limits extensive chain mobility during tensile stress [27,28]. The modest standard deviations throughout most of the strain range signify commendable repeatability, whereas the marginal rise in variability near the peak stress region is ascribed to localized stress concentrations and minor thickness or microstructural discrepancies intrinsic to solution-cast films.

The cross-sectional morphology of the diaphragm, shown in Figure 7b, shows an internal structure with no obvious voids or delamination across the film thickness. The interface appears continuous, suggesting film formation during casting and drying. The internal arrangement is advantageous for diaphragm-based acoustic sensing, as it may facilitate mechanical vibration transfer while reducing internal energy loss. The relatively uniform thickness is consistent with the mechanical response observed in the tensile profile (Figure 7a), confirming the diaphragm’s suitability for vibration-responsive biomedical applications.

Figure 7c delineates the flexibility and mechanical characteristics of the chitosan diaphragm. The diaphragm preserves its overall shape during folding, twisting, and bending, and returns close to its initial configuration after deformation. This behavior suggests elastic recovery and tolerance to repeated mechanical manipulation. Such flexibility is important for wearable and contact-oriented biomedical applications, as diaphragms must conform to the contours of the human body and accommodate continuous low-amplitude deformations during the acquisition of physiological signals [29].

The integrated tensile, morphological, and flexibility characteristics indicate that the chitosan diaphragm exhibits a combination of moderate tensile strength, low strain tolerance, and mechanical robustness. These features are consistent with the mechanical requirements of biomedical acoustic diaphragms.

In addition, the FTIR spectrum (Figure 7d) confirms the chemical structure of the chitosan diaphragm, showing characteristic absorption bands associated with O–H and N–H stretching, amide I and amide II groups, and polysaccharide backbone vibrations. These features are consistent with previously reported chitosan spectra, supporting the successful extraction and processing of the material.

### 3.2. Heart Sound Signal Characteristics at the Aortic Auscultation Site

**Figure 8:**
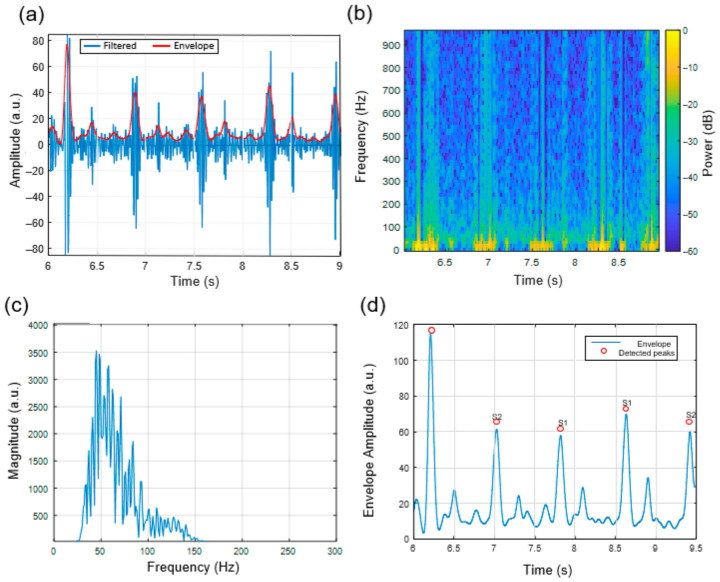
Heart sound signal characteristics recorded at the Aortic auscultation region (right 2nd intercostal space). (**a**) Band-pass filtered waveform (30–150 Hz) with Hilbert-derived envelope showing clearly separated S1 and S2 components. (**b**) Time–frequency spectrogram of the filtered signal demonstrating dominant spectral energy between 40–120 Hz. (**c**) Magnitude spectrum of the last 1 s segment, illustrating peak frequency content typical of the Aortic valve area. (**d**) Smoothed envelope with automatically detected S1 and S2 peaks, confirming stable cycle-to-cycle temporal morphology.

The heart sounds recorded at the aortic region showed identifiable S1 and S2 components which appeared in Figure 8a. The Hilbert-derived envelope facilitated the identification of cardiac events in time, enabling segmentation of cardiac cycles. The spectrogram analysis in Figure 8b showed that most energy was observed between 40 and 120 Hz, consistent with reported acoustic patterns of aortic valve closure [30,31].

The magnitude spectrum (Figure 8c) showed similar spectral peaks across the observed cycles, and automated envelope-based peak detection (Figure 8d) enabled identification of S1 and S2 events. The results indicate that the system can capture repeatable signal patterns, while the chitosan diaphragm does not introduce evident distortion in the observed signals.

### 3.3. Heart Sound Signal Characteristics at the Pulmonic Auscultation Site

**Figure 9:**
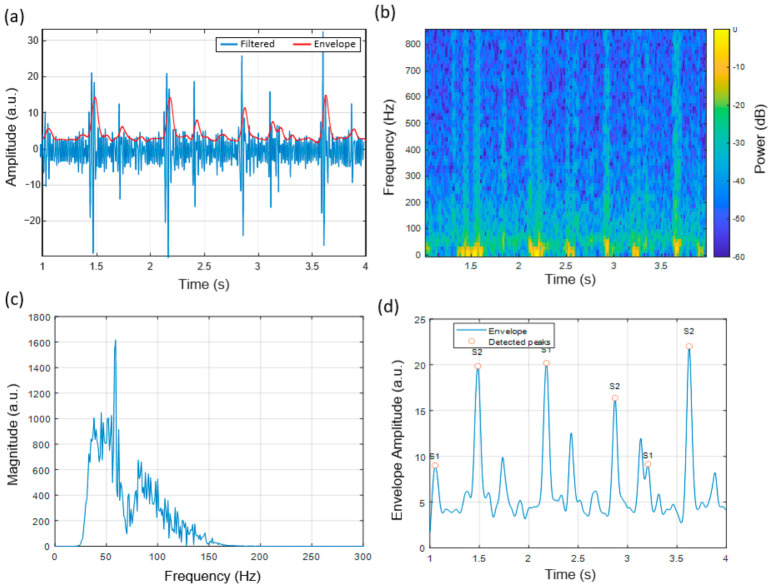
Heart sound signal characteristics recorded at the Pulmonic auscultation region (left 2nd intercostal space). (**a**) Band-pass filtered waveform and envelope, highlighting differences in S1/S2 amplitude patterns compared with the Aortic region. (**b**) Spectrogram showing energy distribution characteristic of pulmonary valve acoustics, with slightly reduced high-frequency content. (**c**) Magnitude spectrum of the final 1 s window, with main peaks concentrated below 100 Hz. (**d**) Smoothed amplitude envelope with identified S1 and S2 peaks, demonstrating consistent segmentation accuracy in the Pulmonic area.

The pulmonic region produced signals with relatively lower amplitude and reduced high-frequency content than the aortic site. The S1/S2 amplitude patterns in Figure 9a illustrate differences observed in the pulmonic region, with identifiable peaks distributed across the cardiac cycles. The waveform shows repeated temporal patterns corresponding to successive cardiac events.

The spectrogram (Figure 9b) showed that higher relative intensity was observed in frequencies below 100 Hz, with a concentration of spectral content in the lower-frequency range and reduced higher-frequency components, consistent with previous phonocardiography research [26]. The magnitude spectrum (Figure 9c) further reflects this distribution, with higher magnitudes concentrated in the lower-frequency range.

Envelope-based segmentation showed similar patterns across different body areas (Figure 9d), with detectable peaks corresponding to S1 and S2 events across multiple cycles.

### 3.4. Heart Sound Signal Characteristics at the Tricuspid Auscultation Site

**Figure 10:**
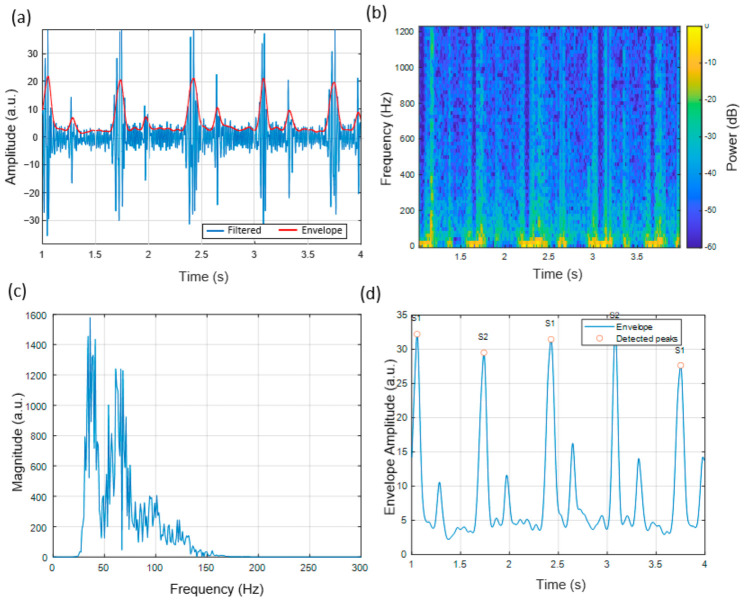
Heart sound signal characteristics recorded at the Tricuspid auscultation region (left lower sternal border, 4th–5th intercostal space). (**a**) Filtered waveform and envelope illustrating lower-frequency, softer acoustic signatures typical of tricuspid valve closure. (**b**) Spectrogram showing broadened low-frequency energy distribution. (**c**) Magnitude spectrum of the last 1 s, with spectral peaks concentrated between 30–80 Hz. (**d**) Smoothed envelope with S1/S2 peak detection, revealing subtle morphological changes specific to the Tricuspid valve region.

Figure 10 shows that heart sounds taken at the tricuspid auscultation region show relatively lower amplitudes and lower dominant frequencies compared to other auscultation sites [6]. The spectrogram (Figure 10b) shows that the spectral content is distributed over a wider low-frequency band. The magnitude spectrum (Figure 10c) shows that prominent spectral components are observed between 30 and 80 Hz.

These observations are consistent with reported phonocardiography characteristics of the tricuspid valve [6]. These sounds have been reported to be characterized by lower amplitude and lower dominant frequency components, consistent with previously reported characteristics of right-sided cardiac sounds [6]. Clinical and signal-processing studies of cardiac auscultation have reported that heart sounds vary in shape and frequency across different regions [26,32].

### 3.5. Heart Sound Signal Characteristics at the Mitral Auscultation Site

**Figure 11:**
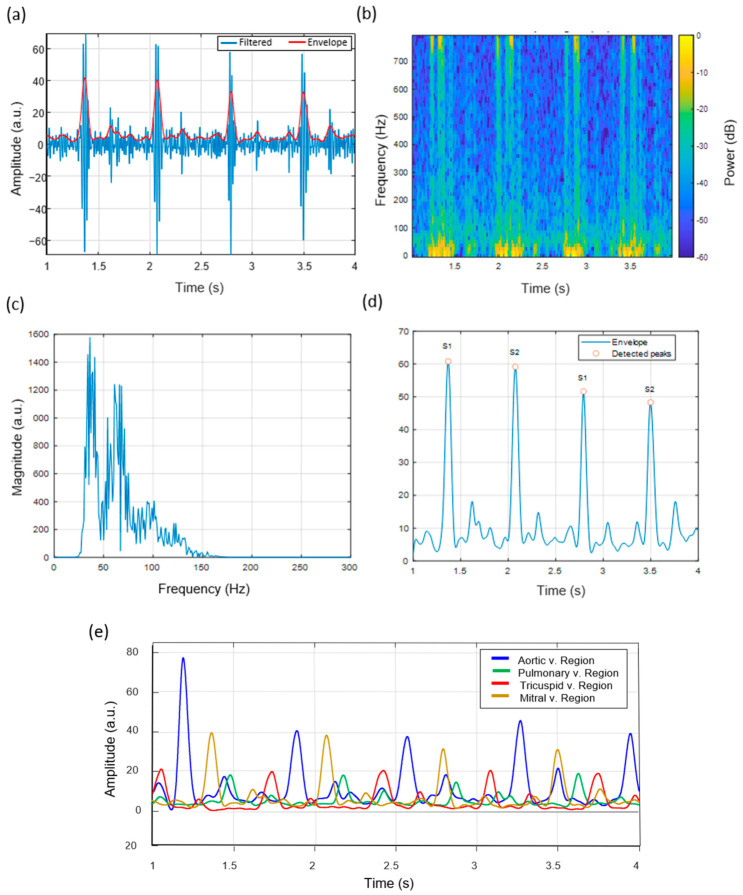
Heart sound signal characteristics recorded at the mitral auscultation region (apex beat). (**a**) Filtered waveform and envelope indicating strong, well-defined S1 events due to proximity to the mitral valve. (**b**) Spectrogram exhibiting dominant mid-frequency energy and sharp S1 intensity. (**c**) Magnitude spectrum of the final 1 s segment, with enhanced spectral components relative to basal regions. (**d**) Smoothed envelope with detected S1 and S2 peaks, demonstrating high S1 amplitude typical of the apical region. (**e**) Envelope amplitude curves from all the 4 valves regions.

Heart sounds captured at the mitral (apical) region display prominent S1 components. Figure 11a shows that the S1 events have relatively higher amplitudes compared to other auscultation regions and well-defined temporal patterns. This is consistent with observations reported in previous studies [26].

The time-frequency analysis (Figure 11b) shows that relatively higher intensity is observed in the mid-frequency range. The envelope-based peak detection results (Figure 11d) show that S1 and S2 events can be identified using the applied signal-processing approach, consistent with reported phonocardiography analysis methods [31].

Finally, Figure 11e presents the superimposed envelope amplitude curves obtained from the four cardiac auscultation regions.

### 3.6. Effect of Humidity on Acoustic Signal Amplitude

To evaluate the effect of humidity on the acoustic response of the chitosan diaphragm, controlled humidity environments were generated using saturated salt solutions. The diaphragm, already mounted in the digital stethoscope assembly, was placed inside a sealed chamber (plastic container) containing saturated aqueous salt solutions to establish specific relative humidity (RH) conditions.

Saturated solutions were prepared by adding excess salt to approximately 50 mL of distilled water until no further dissolution occurred. Magnesium chloride (MgCl_2_), magnesium nitrate (Mg(NO_3_)_2_), and sodium chloride (NaCl) were used to achieve target humidity levels of approximately 30%, 55%, and 75% RH, respectively, consistent with standard humidity control methods based on saturated salts.

For each condition, the assembled stethoscope system was placed inside the chamber and allowed to equilibrate for 1 h to ensure moisture stabilization of the chitosan diaphragm. After conditioning, the system was removed from the chamber and measurements were performed within 3 min to minimize changes in moisture content due to ambient exposure.

This procedure was repeated sequentially for each humidity condition, with reconditioning performed prior to each measurement. During testing, the sensor was subjected to controlled mechanical excitation at approximately 1 Hz, and the resulting signals were recorded for subsequent analysis.

**Figure 12:**
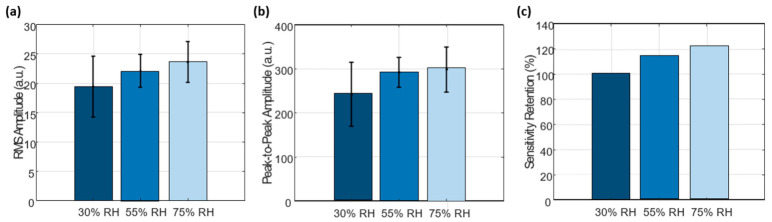
Effect of humidity on acoustic signal amplitude of the chitosan diaphragm. (**a**) Root mean square (RMS) amplitude of the recorded signals under different relative humidity (RH) conditions (30%, 55%, and 75%). (**b**) Peak-to-peak amplitude showing consistent variation with humidity. (**c**) Sensitivity retention (%) calculated relative to the baseline condition (30% RH). Measurements were performed after humidity conditioning using saturated salt solutions, with each condition measured five times (n = 5). Error bars represent standard deviation.

The results (Figure 12) show that the RMS amplitude of the recorded signals increased with increasing relative humidity, indicating a higher signal intensity under more humid conditions. A similar trend was observed in the peak-to-peak amplitude, confirming that the variation in signal magnitude is consistent across different amplitude metrics. In addition, the calculated sensitivity retention exceeded 100% at higher humidity levels. This behavior can be attributed to the humidity-dependent mechanical response of the chitosan diaphragm. As RH increases, the material absorbs moisture due to its hydrophilic nature, leading to partial plasticization and increased flexibility. This enhances the deformation capability of the diaphragm under the same mechanical excitation, resulting in higher vibration amplitudes. Since the sensitivity retention is calculated relative to the baseline condition (30% RH), the increase in signal amplitude at higher humidity levels results in values exceeding 100%. Therefore, the observed behavior reflects a relative enhancement in signal amplitudes rather than an anomaly.

## 4. Discussion and Future Directions

The proposed chitosan-based diaphragm digital stethoscope recorded cardiac acoustic features that are consistent with reported phonocardiography characteristics at all standard auscultation sites [5]. The differences in the amplitude, time structure, and spectral distribution of heart sounds at the aortic, pulmonic, tricuspid, and mitral regions are consistent with reported valve-specific acoustic patterns. These results suggest that key acoustic signal characteristics are retained [26,33].

The flexible chitosan film diaphragm functions as a compliant acoustic interface, providing mechanical conformity to the chest surface. This may allow for the transmission of low-amplitude heart sounds while maintaining mechanical integrity under repeated bending conditions. Polymer-based and flexible diaphragms have been reported to enhance acoustic sensitivity and user comfort, particularly for wearable and handheld auscultation devices [24,29].

In addition, the integration of signal acquisition with automated signal-processing algorithms supports visualization, segmentation, and analysis of heart sounds. These kinds of features are used in modern digital stethoscope platforms, including applications in telemedicine, remote patient monitoring, and medical education [4,6]. Together, these features suggest the potential of the proposed system as a potential alternative concept to traditional stethoscopes, particularly for portable and digitally enabled healthcare applications.

The present study includes certain limitations that should be considered. From a structural standpoint, although the device operates as intended, the use of 3D-printed materials for the housing may not provide the same level of acoustic transmission efficiency as metallic chest pieces used in conventional clinical stethoscopes. Further optimization of the mechanical interface may improve vibration transfer.

From a material perspective, the chitosan used in this study was extracted through a laboratory-scale process and exhibits inherent heterogeneity. The solution contains polymer chains with a broad molecular weight distribution, as well as aggregation and intermolecular interactions associated with its partially ionized nature. As a result, precise quantitative determination of molecular weight and degree of deacetylation (DD) was not achieved, and these properties are interpreted qualitatively based on the observed viscosity and mechanical behavior of the films.

From a system perspective, the current implementation is based on wired data acquisition. While wireless approaches could be explored in future developments, careful design considerations will be required to preserve signal integrity and timing performance [34].

It is also important to note that this work represents a proof-of-concept prototype. The acquired signals show frequency content and amplitude characteristics consistent with expected heart sound behavior [35,36], supporting the feasibility of the proposed approach [28]. Further studies under controlled conditions would be beneficial to extend these findings toward broader applications.

Finally, the results suggest that chitosan is a promising material for acoustic sensing applications. Additional studies focused on its acoustic transmission properties may provide further insight into its potential for bio-based sensing systems.

## 5. Conclusions

This research presents the development of a chitosan-based diaphragm stethoscope prototype through its design process, fabrication stage, and subsequent experimental testing of the signal acquisition system. This study describes the conversion of mealworm biomass–derived chitosan into a flexible diaphragm film, which exhibited measurable elongation properties and mechanical stability under acoustic loading conditions. The diaphragm was integrated into a custom 3D-printed chest piece using piezoelectric sensing to convert thoracic vibrations associated with cardiac activity.

The heart sounds recorded at four standard auscultation sites showed waveform and spectral patterns consistent with reported characteristics of the aortic, pulmonic, tricuspid, and mitral regions. The analysis of time-frequency data, envelope extraction, and automated S1/S2 peak detection demonstrated the capability of the system to segment heartbeats and identify cardiac cycles across the recorded sites.

The results suggest that the proposed biopolymer diaphragm preserves relevant acoustic signal features while providing mechanical flexibility, supporting the potential of biopolymer-based materials for diaphragm applications. The system combines biodegradable chitosan diaphragms with ESP32-based data acquisition and MATLAB-based signal processing to create a digital auscultation prototype with an emphasis on simplified system design and the use of biodegradable materials.

The system design may have potential future applicability in areas such as cardiac signal analysis and medical education; however, further studies and system improvements are required to validate its suitability for these applications.

## Data Availability

The original contributions presented in the study are included in the article, further inquiries can be directed to the corresponding authors.
